# Supplementary material for: Intermittent preventive treatment comparing two versus three doses of sulphadoxine pyrimethamine (IPTp-SP) in the prevention of anaemia in pregnancy in Ghana: A cross-sectional study
Source: PLoS One. 2021 Apr 20;16(4):e0250350. doi: 10.1371/journal.pone.0250350 (PMC8057609; doi:10.1371/journal.pone.0250350)
Supplement: S1 File — (DOCX) [file pone.0250350.s002.docx]

**AVAILABILITY OF DATA AND MATERIAL**

All relevant data are within the manuscript and its Supporting Information files.

**COMPETING INTERESTS**

The authors declare they have no competing interests

**FUNDING**

None

**AUTHORS' CONTRIBUTIONS**

YNA conceived the research idea, designed the study, interpreted the participants data on IPTp-SP dose-dependent efficacy, and drafted the manuscript, SN critically reviewed the manuscript and helped in the revision of the manuscript, RBA analysed and interpreted the data and EOD supervised the study and reviewed the manuscript. All authors read and approved the final manuscript.
