# Supplementary material for: Intermittent preventive treatment comparing two versus three doses of sulphadoxine pyrimethamine (IPTp-SP) in the prevention of anaemia in pregnancy in Ghana: A cross-sectional study
Source: PLoS One. 2021 Apr 20;16(4):e0250350. doi: 10.1371/journal.pone.0250350 (PMC8057609; doi:10.1371/journal.pone.0250350)
Supplement: Attachment — Submitted filename: Response to reviewers.docx [file pone.0250350.s003.docx]

University for Development Studies

School of Public Health

Tamale

14^th^ January, 2021

Dear Editor,

Revision was requested to be made regarding a paper that was submitted to your journal with the topic **“Intermittent Preventive Treatment Comparing Two Versus Three Doses of Sulphadoxine Pyrimethamine (IPTp-SP) in the Prevention of Anaemia in Pregnancy in Ghana: A Cohort Study (Long Title)”**

**We have addressed the concerns raised by the 2 reviewers.**

We thank the reviewers for the generous comments, their time and expertise and have edited the manuscript to address their concerns as shown below;

Reviewer #1: General comments:
This is a manuscript which objective was to compare two versus three doses of Intermittent Preventive Treatment (IPTp) of Sulfadoxine-pyrimethamine (SP) in preventing anemia in pregnant women in Ghana, by Yaa Nyarko et al.
Although the manuscript objective is relevant, some caveats are found in the methods, the results and the conclusion sections as suggested below:

1. Abstract:
Line 25: It is said that “….from a maximum of two…” Actually this statement is not correct, as WHO recommended at least two doses and not only two doses. Thus, the maximum of two is not accurate.

This has been corrected and highlighted in the marked copy of the manuscript.

Line 41: As, this was a cohort study, one is wondering if odds ratio should be used as a measure of the estimate. More details are provided the method section.

Odds ratios (OR) are commonly reported in the medical literature as the measure of association between exposure and outcome and it is applicable in cohort studies as well.

In prospective studies (cohort studies), where the number at risk (number exposed) is available, either RR or OR can be calculated.

Multiple logistic regression, a frequently used multivariate technique, calculates adjusted ORs and not RRs.

2. Introduction:

Line 58: a reference is needed for this sentence. Also, this first sentence should be given after the second sentence which should come first.

The reference has been included

Line 66-67. This sentence is confusing as anaemia in pregnant women is multifactorial, but not saying that “ direct causes of anaemia … are multifactorial…” Also, poverty is not a direct cause of anaemia. Thus, this sentence needs to be clearly written.

This has been corrected and highlighted in the manuscript

Line 93-95: the sentence “they further…….. in pregnancy” is confusing as written. Thus, a clear sentence is needed.

This has been corrected and highlighted in the department

3. Methods:
Line 143: How women in the no IPTp-SP group were followed until 36 weeks from their inclusion. An ethical issue could happen in the context of this study.

What basically was done was that, the pregnant women were not stratified into the three groups at the beginning. They were recruited and their baseline characteristics taken at the first time they were met. Then during delivery another data were taken. The stratification was done at the analysis stage based on the number of SP doses the women had taken

Line 144: It is unclear how IPTp-SP doses is considered as a depending variable.

This was a mistake and it has been corrected


Line 151: is that mean that all women were recruited at 16 weeks gestation? This needs be clarified, as inclusion criteria below is not clear.

This has been clarified in the manuscript as well. The pregnant women were recruited at 16 weeks or more.

Line 154-156: Are these elements part of the inclusion criteria. I wonder if these are not exclusion criteria instead. Thus, the elements of the inclusion criteria are expected.

This error has been corrected and highlighted in the manuscript


Line 167: A precision of 3% was used in the sample size calculation. Can the authors justify why this was used in parallel with high proportion (85%).

The justification has been provided in the manuscript

Line 197: Univariate and multivariate analysis were used to measure our estimate. Was it a logistic regression? Also, as this was a follow-up study, one would wonder if an odds ratio should be used. In addition, one would like to know how variable in the univariate and multivariate models have been selected, as this could change the direction of the results generated in the two models as well as the conclusion provided.

Yes, logistic regression was used in the analysis. How the variables were selected have been clarified in the methodology


4. Results:
Line 205: “Overall……in the study” in fact, all the 1188 were included initially, but not only the 1181 whom completed the follow-up. This in the baseline characteristics suggested in table 1, results should reflect the 1188 and not only the 1181. Also, in table 1, where ever mean is reported, a standard deviation should follow. Eg. Age….

Cross tabulation of socio-demographic and anaemia were performed. Haemoglobin level could not be checked at 36 weeks as per protocol so they were excluded. This people left the community after 1^st^ contact. The standard deviation has been included.

Table 2:
Trimester at 1st ANC: As inclusion criteria was not stated correctly, one would wonder how women included from 25 to term were assessed for their haemoglobin at 36 weeks gestation. Please clarify that.

This has been clarified. Recruitment was at 16 weeks were more. Irrespective of the gestation at which the pregnant woman reported to the hospital, the baseline Hb was checked and at 36 weeks the Hb was checked as well. As per protocol, haemoglobin level is supposed to be checked at registration and at 36 weeks.

Line 247: “the was…..current study” there is a typos error here to check.

This has been corrected in the manuscript

Table 5: Among the variables included in this model, one would like to see the inclusion of other variables such as anaemia at enrolment, trimester or gestational age of SP uptake, ITN use, ect… Authors should provide the criteria for a variable to be included in the two models. With the absence of important variables in the models, one would be cautious about the conclusion provided for this study. Thus, the discussion section could be adjusted accordingly.

Other variables have been included in the as well. It is now Table 3.

Table2: Less than 3 doses where protective of anaemia in the univariate model although borderline significant. However, in the multivariate model, this is considered as a risk factor. Can the authors provide this justification?

This has been corrected as well in the manuscript

Conclusion:
In this section, results in term of numbers are not expected. Eg 55.8%. 62.6%, ect…. These are already available in the results section. Also, explanation (eg line 386-line 388) is not supposed to be given in that section as discussion section is allocated for that.

This has been corrected in the conclusion.

Line 391-392: The conclusion could be reviewed after controlling for other additional variables in the regression model.

This has been corrected as well

Reviewer #2: The purpose of this article is to test the effectiveness of the new policy on intermittent preventive treatment with Sulfadoxine-Pyrimethamine in Ghana to prevent anemia in pregnant women, and to determine the optimal dosage of protection for pregnant women against anemia.

The subject is not without interest, but the manuscript in its current form suffers from major drawbacks which seriously compromise the reliability of the conclusions drawn by the authors:

The study is presented as a cohort study but it is not clear how the data were processed longitudinally. The main outcome is anemia at 36 weeks gestation, which is a very partial summary of anemia during pregnancy, and the choice of this outcome is not explained

It is not clear whether pregnant women are recruited at the first antenatal visit or later during pregnancy.

This has been addressed in the manuscript

Longitudinal study is useful in examination of the natural course of a disease over time, without systematically manipulating the state of that disease.

The method of measuring hemoglobin level is not mentioned.

This has been included in the manuscript

It is not mentioned if some women received doses of IPT after 36 weeks of pregnancy (which would of course compromise the conclusion about the impact of IPTp on anemia at 36 weeks)

The protocol is to check the Hb at 36 weeks of gestation and IPT is not taken after 36 weeks

No comparison between women who received less than 2 doses and those who received 3 or more doses was shown, which makes it difficult to assess the potential study biases.

There is comparison between those who took the IPTp-SP; the stratifications were those who did not take SP, those who took 1 or 2 doses and those who took 3 or more has been shown in Tables 2 and 3

The manuscript presents the calculation of a sample size without the assumption on which this calculation is based being clearly explained, and without any evident link to the stated objectives of the article.

This has been corrected and clearly stated in the manuscript

Table 4 is presented as a multivariate logistic regression but no details are given neither on the adjustment variables nor on the method of variable selection

This has been included in the manuscript and highlighted in the methodology section

The authors mention a design-effect (intra-hospital correlation) without showing how the statistical analysis takes this element into account. The draft does not seem to have been reread in a rigorous manner, and several inconsistencies remain:

- the paragraphs inclusion criteria and exclusion criteria are identical (and only concern the exclusion criteria),

- line 244 Table 4 is confused with table 5

- etc…

This has been addressed and highlighted in the manuscript

In summary, the level of evidence of the conclusions is very low.

This has been worked on and highlighted in the manuscript

We believe the manuscript is now ready for publication in the PLOS ONE journal.

Yaa Nyarko Agyeman (Ph.D)

Lecturer

Dept of Population and Reproductive Health

On behalf of all authors
