# Supplementary material for: Intermittent preventive treatment comparing two versus three doses of sulphadoxine pyrimethamine (IPTp-SP) in the prevention of anaemia in pregnancy in Ghana: A cross-sectional study
Source: PLoS One. 2021 Apr 20;16(4):e0250350. doi: 10.1371/journal.pone.0250350 (PMC8057609; doi:10.1371/journal.pone.0250350)
Supplement: Attachment — Submitted filename: Response to reviewers 2.docx [file pone.0250350.s004.docx]

University for Development Studies

School of Public Health

Tamale

30^th^ March 2021

Dear Editor

Revision was requested to be made regarding a paper that was submitted to your journal with the topic **“Intermittent Preventive Treatment Comparing Two Versus Three Doses of Sulphadoxine Pyrimethamine (IPTp-SP) in the Prevention of Anaemia in Pregnancy in Ghana: A Cross-sectional study (Long Title)”**

**We have addressed the concerns raised by the reviewer.**

We thank the reviewers for the generous comments, their time and expertise and have edited the manuscript to address their concerns as shown below.

Reviewer #1: General comments:
This is a very good and well written manuscript which aims to compare two versus three doses of intermittent preventive treatment using sulfadoxine-pyrimethamine to prevent maternal anaemia during pregnancy. However, there is a need of clarifying some points in order to increase the quality of this work.


1. Abstract:

Line22-23: In fact, this recommendation started from 2012 with a policy brief but not in 2014 as indicated by the authors.

This correction has been done.

2. Background:
Line 72-74: The sentence “The science of IPTp……prevented” is not from the author indicated in the manuscript. Please make necessary correction

The author has changed.

Line 77: The authors indicated that 60% of anaemia was observed in studies from references 1, 14-16 in Tanzania. After verification of the references, the 60% is not found. Please verify or rewrite this sentence according to the reality.

The 60% was for Nigeria. No percentage was stated for Tanzania; however, we have included the prevalence for Tanzania now.

Line 82-83: the sentence “The prevalence…. transmission areas” can the authors indicate the reference of this? Also, is that sentence correct? One would wonder if the authors want to say 61% among non-users versus 56% among users.

These corrections have been made


3. Methods:
Sample size calculation

Line 161-162: Can the authors provide the rational of using the precision of 3% given the high proportion of 85% considered.

This is the reason for using a precision of 3%. This is part of a bigger study and one of the outcomes that was of interest was stillbirth, which is a rare occurrence. The sample size was increased to help get the needed information as well and to help in generalization of the study as well.

Also, not sure if the proportion of 85% in anticipated target should be used. Instead, proportion is supposed to come from previous studies.

This has been corrected and referenced appropriately.

Line 196: as malaria seasonality has been considered, performing the analysis by season is preferable to take this component into account.

This statement has been corrected.

Line 207-209: As this was a cohort study according to the authors, one would wonder if Odds ratio should be used to estimate the association between predictors and outcome. Can the authors provide argument about not using risk ratio?

Thank you for the comment. The design used has been corrected.

4. Results:

Line 271-272: the sentence: “prevalence of …….pregnancy”. Can the authors provide the over form of intervention used during pregnancy?

The statement was not too clear however, the other interventions have been included

Table 3: Can the authors provide the meaning of MPs under the table?

It has been written in full, Malaria parasites

5. Discussion:

Line 353: The sentence: “It was discovered…” Is this a discover? It is probably an observation.

It has been changed to an observation

Line 355: The rates of anaemia (63.5% versus 61.5%) provided are said to be different by the authors. One would wonder if this is correct, as these are really similar.

This correction has been done

Line 363: The authors suggested that other factors than malaria may be responsible of increasing the risk of anaemia. Can the authors provide these factors?

The other factors have been provided.

Line 374-375: As 62% is different from 73.5% and 47.2%, what is the supportive argument of this consistency suggested?

The grammar has been corrected

Line 381-383: The prevalence of anaemia provided in different doses of SP seem to indicate a protection against anaemia. However, the conclusion provided by the authors suggest non protective effect of SP on anaemia. Can the authors provide argument to explain this discrepancy between their conclusion and what is observed here (line 381-383)?

This has been done please

Line 412-413: It is said that malaria in this setting is not the cause of anaemia. This statement is questionable, as malaria can always cause anaemia, although it is not main contributor in this setting.

This has been corrected

Line 470: limitations
One limitation in this study could be the fact that no parasitemia results were available at 36 weeks to better explain the relationship between IPTp-SP and anaemia.

Parasite results were available and were part of the adjusted model. That was the MPs…I have written it in full. The effect of the malaria infection was considered, and it was found that, malaria during late gestation significantly influenced anaemia.

Conclusion:
Line 480-481: The two sentences can be combined in one.

It has been done

Line 488-489: “….the need ……the odds of anaemia in pregnancy…”. This is not demonstrated in this study.

it has been corrected.

Line 491-492: The sentence “further research….part if Ghana” as this is a recommendation, it is important to provide details on the deficiencies related with the intervention used.

Thais has been done.

We believe the manuscript is now ready for publication in the PLOS ONE journal.

Yaa Nyarko Agyeman (Ph.D)

Lecturer

Dept of Population and Reproductive Health

On behalf of all authors
